# Etude rétrospective sur 60 cas de rupture utérine du centre de maternité de Monastir, Tunisie

**DOI:** 10.11604/pamj.2024.47.83.42188

**Published:** 2024-02-26

**Authors:** Imen Ben Farhat, Olfa Zoukar, Malak Medemagh, Wiem Ben Slamia, Amina Mnajja, Haifa Bergaoui, Ahmed Hajji, Mouna Gara, Dhekra Toumi, Raja Faleh

**Affiliations:** 1 Université de Monastir, Faculté de Médecine de Monastir, Monastir, Tunisie,; 2 Service de Gynécologie Obstétrique du Centre de Maternité et de Néonatologie de Monastir, Monastir, Tunisie,; 3 Service d'Anesthésie Réanimation du Centre de Maternité et de Néonatologie de Monastir, Monastir, Tunisie

**Keywords:** Rupture utérine, utérus cicatriciel, utérus non cicatriciel, déhiscence, pronostic, Uterine rupture, scarred uterus, unscarred uterus, dehiscence, prognosis

## Abstract

La rupture utérine est une complication obstétricale redoutable. Le but de notre travail est d'étudier les caractéristique épidémiologiques, les éléments de pronostic maternel et fœtale ainsi que les différentes modalités thérapeutiques de la rupture utérine survenue sur utérus sain et cicatriciel. Nous avons mené une étude rétrospective monocentrique descriptive et analytique, portant sur 60 cas de rupture colligées au service de gynécologie obstétrique du centre de maternité et de néonatologie de Monastir, s'étalant de 2017 jusqu'en 2021. Les patientes ont été classées selon la présence ou non d'une cicatrice utérine. Soixante patientes ont été incluses. La majorité des cas de rupture étaient survenus sur utérus cicatriciel (n=55). Le signe clinique le plus retrouvé a été l'anomalie de rythme cardiaque fœtal. Aucun décès maternel n'a été enregistré et le taux de mortalité périnatale a été de 11%. Nous avons trouvé un indice de masse corporelle (IMC) moyen et un taux de macrosomie fœtale et une parité moyenne significativement plus élevés dans le groupe utérus sain que celui dans le groupe utérus cicatriciel (p=0,033, 0,018 et 0,013 respectivement). Les complications maternelles étudiées (hémorragie du post partum, hystérectomie, transfusion sanguine, hospitalisation prolongée) ont été significativement plus fréquentes en cas de rupture utérine (RU) sur utérus sain (p=0,039; p=0,032; p=0,009; p=0,025 respectivement). La rupture utérine est un accident obstétrical mettant en jeu le pronostic fœtal et maternel. L'anomalie de rythme cardiqaue d’un foetus (RCF) est le signe révélateur le plus fréquemment trouvé. Le traitement est conservateur dans la majorité des cas. Le pronostic est meilleur en cas d'utérus cicatriciel.

## Abstract (en)

Uterine rupture is a life-threatening obstetric complication. The purpose of this study was to investigate the epidemiological features, maternal and foetal prognosis and different treatment options for uterine rupture in healthy and scarred uteri. We conducted a retrospective monocentric descriptive and analytical study of 60 cases of uterine rupture collected in the Department of Gynaecology-Obstetrics of the Center of Maternity and Neonatology, Monastir, from 2017 to 2021. Patients were classified according to the presence or absence of a uterine scar. Sixty patients were enrolled in the study. The majority of cases of rupture occurred in patients with scarred uterus (n=55). The most common clinical sign was abnormal foetal heart rate. No maternal deaths were recorded and perinatal mortality rate was 11%. Mean BMI, fetal macrosomia rate and mean parity were significantly higher in the healthy uterus group than in the scarred uterus group (p=0.033, 0.018, and 0.013, respectively). The maternal complications studied (post-partum haemorrhage, hysterectomy, blood transfusion, prolonged hospitalisation) were significantly more frequent in patients with unscarred uterine rupture (p=0.039; p=0.032; p=0.009; p=0.025 respectively). Uterine rupture is a life-threatening obstetrical event for the foetus and the mother. Fetal heart rate abnormality is the most common sign associated with uterine rupture. Management is based on conservative treatment in most cases. Patients with scarred uterus have a better prognosis.

## Introduction

La rupture utérine est une complication obstétricale redoutable, grevée d'une lourde mortalité materno fœtale. Elle se définie comme une solution de continuité non chirurgicale de la paroi utérine survenue au cours de la grossesse ou pendant le travail [1]. Son pronostic dépend étroitement de la précocité et la qualité de la prise en charge. Devenue exceptionnelle dans les pays développés avec une prévalence de 0,5/10000 à 7,9/10000 naissances [2], la rupture utérine reste l'apanage des pays sous médicalisés. L'inflation des taux de césariennes au cours de ces 20 dernières années et le recours à une épreuve utérine ont contribué à l'augmentation du risque de la rupture utérine. En Tunisie, le taux de césarienne ne cesse d'augmenter atteignant 43,2% en 2018 [3], ceci nous laisse nous interroger sur le risque de la rupture utérine qui lui est associé. Au cours de ce travail nous avons étudié les caractéristique épidémiologiques, les éléments de pronostic maternel et fœtale ainsi que les différentes modalités thérapeutiques de la rupture utérine survenue sur utérus sain et cicatriciel.

## Méthodes

Nous avons mené une étude rétrospective descriptive et analytique, portant sur une série consécutive de 60 cas de rupture colligées au service de gynécologie obstétrique du centre de maternité et de néonatologie de Monastir (CMNM), s'étalant sur une période de 5 ans du 01/01/2017 au 31/12/2021.

N'ont pas été incluses dans cette étude, tous les cas d'amincissement considérable d'une cicatrice utérine lors d'une césarienne itérative ou d'une révision utérine après accouchement par voie basse ainsi que les déchirures limitées au col utérin, Les perforations utérines survenant lors d'un avortement et les cas de placenta accréta. Nous avons exclu de notre étude les patientes ayant eu des dossiers incomplets ou inexploitables.

Le recueil des données a été réalisé de façon rétrospective à partir des dossiers médicaux des patientes et des cahiers de compte rendu opératoire. L'analyse statistique était réalisée à partir des logiciels Microsoft Office Excel 2019 et IBMSPSS.

Les statistiques étaient descriptives et analytiques. Les variables quantitatives étaient décrites par leur moyenne, médiane, maximum, minimum et les variables qualitatives par leur effectif et pourcentage. Les tests utilisés étaient: le Test de Student pour les comparaisons de moyennes entre deux groupes en cas de distribution normale et respectivement les tests non paramétriques de MANN WITNEY dans le cas contraire et le Test du Khi-deux pour l'indépendance de deux variables qualitatives. Le seuil de signification p a été fixé à 5%.

## Résultats

**Figure 1:**
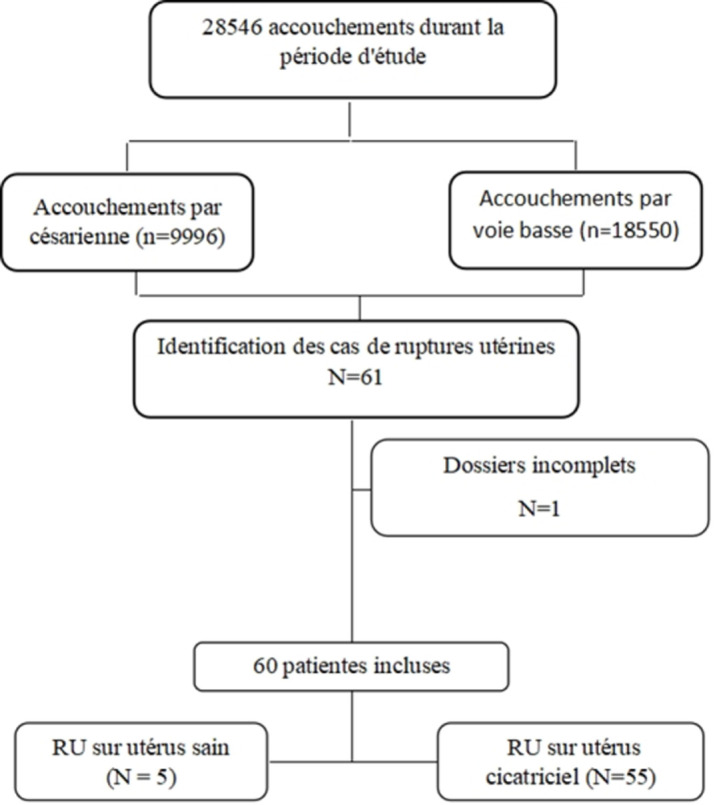
diagramme de flux de sélection des patientes

Durant la période d'étude, 28546 accouchements ont eu lieu (en moyenne 5709 accouchements par an). Nous avons recensé 61 cas de rupture utérine durant la même période soit un taux de 2,13%. Les dossiers complets retenus pour les analyses étaient ceux de 60 patientes (Figure 1). L'âge moyen était 30,88 ± 0,635 ans avec des extrêmes de 18 et 42 ans. La rupture utérine était enregistrée dans 91,6% des cas sur utérus cicatriciel et dans 75% des cas chez des patientes ayant un utérus uni cicatriciel (Tableau 1). La majorité des cas de RU ont été découvert à terme (91,6%).

**Tableau 1:** caractéristiques épidémiologiques et cliniques des patientes

Caractéristiques		Utérus sain (N=5)	Utérus cicatricial (N=55)
Age moyen des patientes		30,80 ± 2,083	30,89 ± 0,672
Age gestationnel moyen		40,3 ± 0,62	39,19 ± 0,22
Parité moyenne		3,6±0,51	2,47 ± 0,089
IMC moyen		34,55 ± 3,03	28,43 ± 0,57
Nombre de césarienne précédente	0	5	0
	1	0	45(82%)
	>1	0	10(18%)
ATCDS de myomectomie		0	7(12,72%)
Grossesse multiple		0	2(3,36%)
Bassin limite		1(20%)	8(14,54%)
Présentation dystocique		0	7(12,8%)
Hydramnios		0	2(3,6%)
Macrosomie (poids fœtal > 4KG)		3(60%)	9(16%)
Signes cliniques	Anomalie de RCF	2(40%)	29(52,7%)
	Métrorragie	2(40%)	10(18%)
Moment de découverte	En dehors du travail	0	10(18,2%)
	Au cours du travail	3(60%)	42(76,3%)
	En post partum	2(40%)	3(5,5%)
Type de rupture	Complète	4(80%)	19(34,5%)
	Incomplète	1(20%)	36(65,5%)
Siège de RU	Segmentaire	0	43(78,2%)
	Corporéale	100%	12(21,8%)

Le signe clinique le plus trouvé était l'anomalie de RCF pour les ruptures sur utérus sain et cicatriciel. La majorité des RU était découverte pendant le travail soit 78,1% du taux total des RU (rupture utérine) et 71% des cas de RU sur utérus cicatriciel. La rupture était incomplète dans 61,7% des cas et segmentaire dans 71,6% des cas. Les ruptures sur utérus sain étaient survenues à dilatation complète et en post partum dans 80% des cas (Tableau 2). Le traitement a été conservateur dans la majorité des cas. Une hystérectomie a été réalisée dans 4 cas. Aucun décès maternel n'a été enregistré et le taux de mortalité périnatale était de 11%. Le score d'Apgar était inférieur à 7 dans 30% des cas. La médiane de la parité des patientes dans le groupe utérus sain a été significativement plus élevée que celle dans le groupe utérus cicatriciel (p = 0,013).

**Tableau 2:** caractéristiques du travail et du mode d'accouchement

Caractéristiques		Utérus sain (N=5)	Utérus cicatricial (N=55)
Travail Spontané		1	44
Travail Induit		4	11
Mode de déclenchement	DPIO	1	6
	Misoprostol	2	0
	Dinoprostone	1	0
	SEA	0	5
Mode d'accouchement	AVB normal	2	3
	Forceps	0	2
	CS en urgence	3	50
Perfusion d'ocytocine		3	0
Poids fœtal Moyenne (gramme)		4000 grammes	3200 grammes
Durée moyenne de la phase de Latence		10,4 heures	7,9 heures
Durée moyenne de la phase active		0,57 heure	2,8 heures
Type de PEC	Suture simple	2	45
	Suture +LT	1	8
	Hystérectomie	2	2
Lésions associées	Plaie vésicale	1	5
	Plaie du col	3	2
	Plaie du vagin	4	0

Concernant les facteurs de risque, nous avons trouvé un IMC moyen et un taux de macrosomie fœtale significativement plus élevés dans le groupe utérus sain que celui dans le groupe utérus cicatriciel (p=0,033 et 0,018 respectivement). La rupture était survenue à un âge gestationnel plus précoce en cas utérus cicatriciel en comparaison avec l'utérus sain, la différence n'a pas atteint une valeur significative (p=0,16). Nous avons également trouvé une différence significative dans le type de la lésion (complète ou incomplète) entre les deux groupes avec 65,5% des RU incomplètes dans le groupe cicatriciel contre 20% dans le groupe sain (p=0,045). Le siège de la rupture en cas d'utérus cicatriciel était corporéal ou segment- corporéal dans 12 cas, soit 21,8%. En revanche, ce taux a été de 100% dans le cas d'utérus sain et la différence était statistiquement significative.

Concernant les caractéristiques du travail, la durée de la phase active a été significativement plus courte dans le groupe utérus sain que celui d'utérus cicatriciel. Le travail était induit dans 80% des cas de RU sur utérus sain contre 20% des utérus cicatriciels (p=0,043).

Les complications maternelles étudiées (hémorragie du post partum, hystérectomie, transfusion sanguine, hospitalisation prolongée) ont été significativement plus fréquentes en cas de RU sur utérus sain (p=0,039; p=0,032; p=0,009; p=0,025 respectivement).

Dans le groupe d'utérus cicatriciel, le pronostic fœtal a été moins grave. En effet dans 75% des RU sur utérus cicatriciels, le score Apgar a été supérieur à 7 contre 20% seulement dans le groupe utérus sain. La différence était statistiquement significative avec p=0,01 (Tableau 3).

**Tableau 3:** comparaisons des caractéristiques épidémiologiques, cliniques anatomiques et pronostiques des RU entre utérus sain et cicatriciel

Caractéristiques		Utérus sains N=5	Utérus cicatriciels N=55	p
Age moyen des patientes		30,80 ± 2,083	30,89 ± 0,672	0,658
Age gestationnel moyen		40,3 ± 0,62	39,19 ± 0,22	0,164
Parité moyenne		3,6 ± 0,51	2,47 ± 0,089	0,013
Hydramnios (N)		0	2	0,66
ATCDS de myomectomie		0	7(12,72%)	0,4
IMC moyen		34,55 ± 3,03	28,43 ± 0,57	0,033
Macrosomie (poids>4kg)		60%	16%	0,018
Type de rupture utérine	incomplète	1(20%)	36(65,5%)	0,045
	complète	4(80%)	19(34,5%)	
Déclenchement du travail		80%	20%	0,043
Durée moyenne de la phase active		0,57 ± 0,2	2,86 ± 0,54	0,000
Complications materno fœtales	Hémorragie du post partum	60%	14,5%	0,039
	Hystérectomie	40%	3,6%	0,032
	Transfusion sanguine	60%	20,0%	0,009
	Durée d'hospitalisation >5jr	80%	25,4%	0,025

## Discussion

L'incidence de la rupture utérine relative à notre service était 21/10 000 accouchements. La fréquence des ruptures utérines varie d'un pays à l'autre. Alors qu'elle peut atteindre des taux assez bas dans les pays médicalisés: 1.6/10000 en Italie [4] et 1.96/10000 en chine [5], la fréquence de RU dans d'autres pays sous médicalisés peut atteindre des taux effrayants de l'ordre de 16% [6]. Cette disparité est le reflet de la différence du niveau de développement socio sanitaire, de la qualité des soins obstétricaux et de l'hétérogénéité de la définition de la RU au sein des séries, certains ayant exclu les RU incomplètes ou déhiscence.

L'utérus cicatriciel est un facteur de risque majeur de la rupture utérine. Dans les pays développés, sa part parmi les RU varie entre 70 et 90% [7]. En revanche, ces fréquences sont relativement plus basses en Afrique noire entre 12 et 41% [6]. Cette constatation ne s'explique pas par la rareté de cette complication sur utérus cicatriciel; mais plutôt par la plus grande fréquence de RU sur des utérus sains épuisés par des grossesses répétées avec des mauvaises conditions de prise en charge. Dans notre série, 91.7% de l'ensemble des RU ont été survenus sur utérus cicatriciel.

L'épreuve utérine est facteur de risque de RU [8]. Dans notre série le taux de RU en cas d'une épreuve utérine a été de 63% des cas. L'âge maternel moyen trouvé dans notre série a été de 30,80 ± 0.635. Filho [8] a trouvé un risque de RU élevé chez des femmes âgées de plus de 30 ans. En revanche certains auteurs en Afrique noir rapportent un maximum de RU chez des femmes âgées de moins de 30 ans. Ces constatations peuvent être contribuées à l'environnement socio culturel dans ces pays incitant les femmes à se marier à un âge très précoce.

La multiparité est généralement considérée comme un facteur de risque de RU à travers les changements histologiques de muscle utérin [9]. Cependant la part de la multiparité est variable selon les auteurs. Ceci peut être expliqué par la variation de taux des multipares dans les populations générales, des autres facteurs de risque et part la présence surtout d'une cicatrice utérine. Dans notre série ce facteur ne s'est concrétisé que dans le groupe utérus sain avec un taux de 60% alors que dans le cas de l'utérus cicatriciel les multipares n'ont pas représenté que 5.5%. La RU peut survenir à tout âge gestationnel mais survient surtout en fin de grossesse et au cours du travail indépendamment de la présence ou non d'une cicatrice utérine [7].

Dans notre série La RU a été à terme dans 91,7% des cas. La rupture était produite au cours de travail dans 87,2% du nombre total des RU à terme et dans 96% des cas des utérus cicatriciels. Ces résultats concordent avec la série de Zwart [10]. En effet parmi 210 cas de RU 171 cas soit 81.4% sont produite au cours de travail dont: 73% sont survenus au cours de la première phase de travail, 18,1% au cours de la 2^e^phase de travail et 8,8% pendant la phase de latence. Les anomalies de RCF étaient les signes le plus fréquemment trouvés chez les parturientes (31/60). Dans la littérature, ces anomalies de tracé sont retrouvées dans 55 à 90% des RU survenues sur utérus cicatriciel [10] indépendamment de modifications de l'activité utérine.

Une douleur abdominopelvienne persistante et d'apparition secondaire doit alerter. Elle peut être présente dans 50 à 70% des cas [10] et [11]. La métrorragie est un signe classique mais n'est pas constant [12]. D'autre signes peuvent être associés à une RU tel que la modification de la dynamique utérine comme l'indique Arulkumaran [13]. La rupture sur utérus sain présente des symptômes hétérogènes et non spécifiques entrainant un retard fréquent de prise en charge et des complications plus graves. Une métrorragie installée de fin de travail ou en post partum immédiat doit alerter. Wang [14] a trouvé dans sa série de RU sur utérus sain la présence d'anomalies de RCF dans 80% des cas.

Concernant la thérapeutique, notre attitude a fait appel à un traitement chirurgicale encadré par une réanimation pré, per et post opératoire afin d'assurer l'hémostase. Un traitement conservateur a été réalisé dans 93,3%. Cette fréquence s'approche des résultats guyot [15]. Ceci peut être expliqué par la rapidité de prise en charge et la nécessité de préserver la fertilité chez les jeunes femmes à faible parité dès que possible. La mortalité maternelle dans notre série a été de 0%. Ceci témoigne de l'amélioration de la prise en charge obstétricale et du plateau technique adéquat. Ces résultats concordent avec les pays à haut niveau socioéconomique où les taux de mortalités sont faibles comme les études suivantes: Chang en 2020: 0% [16]; et Zwart en 2010: 0% [10].

D'autres études estiment néanmoins que la mortalité maternelle n'est pas nulle. C'est le cas des pays à faible développement socio sanitaire où on enregistre des taux de: 11,26% dans l'étude menée en Côte d'Ivoire par d' Abauleth *et al*. [17], et 17,1% dans celle réalisée au centre d'Afrique par Sepou [18]. La mortalité périnatale varie selon les séries entre 8,7 et 16% [9,18]. Le pronostic maternofœtal est différent qu'il s'agit d'un utérus sain ou cicatriciel. Nos résultats sont comparables à ceux de zwart [10]. En effet, les complications materno fœtales sont plus fréquente en cas d'utérus sain. Ceci est dû probablement à l'invraisemblance de diagnostic, le retard de prise en charge et l'étendue des lésions.

## Conclusion

La rupture utérine est une urgence médico chirurgicale et obstétricale rare mais grave. Dans la majorité des cas, la rupture se produit à terme et au cours du travail, cependant, il faut toujours y penser même en absence des facteurs de risque, en dehors du travail et dans la période du post partum. L'anomalie de RCF est le signe révélateur le plus fréquemment trouvé. La gravité de cette complication implique la vigilance de l'équipe obstétricale pour assurer une prise en charge rapide, sans délai encadrée par une réanimation pré, per et post opératoire. Le traitement est conservateur dans la majorité des cas. Le pronostic maternofoetale est meilleur en cas d'utérus cicatriciel.

### Etat des connaissances sur le sujet

- *La rupture utérine est une complication obstétricale redoutable grevée d'une lourde mortalité maternelle et fœtale plus fréquente en cas d'utérus cicatriciel;*
- *Les données publiées dans la littérature montrent un pronostic materno fœtal meilleur en cas d'utérus cicatriciel*.

### Contribution de notre étude à la connaissance

- *La rupture utérine est plus fréquente sur utérus uni cicatriciel et se produit surtout à terme et au cours du travail;*
- *Les anomalies de RCF sont les signes révélateurs le plus fréquents de rupture utérine en présence ou non de cicatrice utérine;*
- *L'obésité, la macrosomie, le travail rapide et le déclenchement du travail sont des facteurs de risques significativement plus fréquents en cas d'utérus cicatriciel*.
